# Entropy-based dynamic graph embedding for anomaly detection on multiple climate time series

**DOI:** 10.1038/s41598-021-92973-8

**Published:** 2021-07-05

**Authors:** Gen Li, Jason J. Jung

**Affiliations:** Department of Computer Engineering, Chung-Ang University, 84 Heukseok-ro, Dongjak-gu, Seoul, 06974 Republic of Korea

**Keywords:** Computer science, Information technology, Scientific data

## Abstract

Abnormal climate event is that some meteorological conditions are extreme in a certain time interval. The existing methods for detecting abnormal climate events utilize supervised learning models to learn the abnormal patterns, but they cannot detect the untrained patterns. To overcome this problem, we construct a dynamic graph by discovering the correlation among the climate time series and propose a novel dynamic graph embedding model based on graph entropy called EDynGE to discriminate anomalies. The graph entropy measurement quantifies the information of the graphs and constructs the embedding space. We conducted experiments on synthetic datasets and real-world meteorological datasets. The results showed that EdynGE model achieved a better F1-score than the baselines by 43.2%, and the number of days of abnormal climate events has increased by 304.5 days in the past 30 years.

## Introduction

Climate events commonly include meteorological conditions such as precipitation, water vapor pressure, atmospheric temperature, and humidity. Abnormal climate event is that some meteorological conditions are extreme in a certain time interval. It endangers the balance of the natural ecosystem and threatens humans’ survival. Abnormal climate event detection can help people analyze the patterns of the abnormal meteorological and extract useful features from these patterns^1^. As a research issue of data mining, anomaly detection has been widely applied to abnormal climate event detection. The object of anomaly detection is to identify the data that are significantly different from most observations.

Existing data analysis methods for anomaly detection are based on machine learning models^2^. The supervised learning methods learn the patterns of abnormal climate events and fit the non-linear models. These methods require that the data has been labeled as anomalies. The semi-supervised learning methods utilize a few labeled data to fit the models and detect the abnormal climate events on the unlabeled data. These methods are commonly constructed by using the autoencoder. If the loss of the data point is greater than the threshold, the data point is detected as an anomaly. These methods are impossible to detect the anomaly without untrained patterns. Climate events comprise the multiple meteorological data, and the correlation between these data also plays an essential role in anomaly detection. To address this problem, we propose using a graph to model the correlation among multiple time series. Graph model integrated dynamical analysis has been demonstrated as an effective tool in data mining fields and industrial processing monitoring, such as seen in the papers^3,4^. They proposed a graph-based change detection for monitoring the statement of machines and diagnosed the faults by detecting the changes in the dynamic graph constructed using the mechanical vibration signals. In our issue, we aim to detect an abnormal graph from the dynamic graph instead of detecting when the dynamic graph changes.

To solve this problem, we propose to construct a dynamic graph by discovering the correlation between the meteorological time series in the previous research^5^. The idea mainly consists of three steps. Firstly, the dynamic graph is constructed by identifying the spurious relationship between meteorological data in which two correlation data are not causally related^6^. The causation has been widely analyzed on multiple time series such as the paper^7^ applied the causation from the time series to the earth sciences to detect and attribute climate change. The vertices of the graphs indicate meteorological data, whereas the edges indicate the spurious relationship between two vertices. Since there are some limitations to detect anomaly only by using the adjacency matrices of the graph, we calculate the graph entropy using the spurious correlation coefficient to measure the similarity between two graphs. Then, we propose a dynamic graph embedding model based on graph entropy to construct an embedding space for discriminating the anomaly. Finally, we apply the existing outlier detection methods on the embedding space to detect the anomaly. The abnormal climate event is detected as a graph in a certain time interval where the entropy is different from most other graphs, which is defined as follows.

### Definition 1

(*Abnormal climate event*) An abnormal climate event is defined as a graph $G_i$ in the *i*-*th* time interval in which the weights of the edges are significantly different from those in other time intervals. It is formulated as $|e(G_i) - e(G_{i-1})| > \theta$ or $|e(G_i)-e(G_{i+1})| > \theta$, where $e(G_i)$ is the entropy of the climate event $G_i$ and $\theta$ is the threshold for detecting the abnormal climate event.

**Figure 1:**
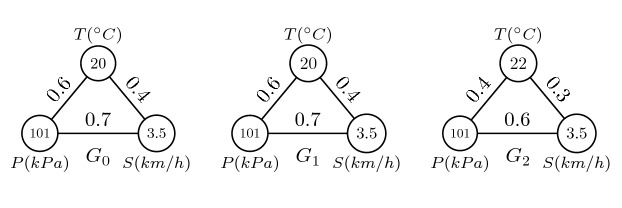
Example of an abnormal climate event.

Figure 1 shows a toy example of the climate events in three time intervals by using the synthetic data where *T* indicates temperature, *P* indicates pressure, *S* indicates wind speed, $G_i$ indicates the climate event in the *i*-*th* time interval, and the weight of an edge indicates the spurious correlation coefficient. The temperature at $G_2$ increases by 2 $^\circ$C, which leads to a difference in the weights of edges and those of the other two time intervals. This indicates that the climate event at $G_2$ is abnormal.

The main contributions of this work are as follows.

- We present the graph entropy to measure the information of climate events, which is calculated using the spurious correlation coefficient.
- We propose an entropy-based dynamic graph embedding model (EDynGE) to cluster the climate events. The abnormal climate events are far from the most observations and can be detected by applying an outlier detection method.
- We conducted experiments on synthetic datasets and real meteorological datasets. The results showed that the proposed method achieved better performance on the two kinds of the datasets than the other dynamic graph embedding models, and the days of the abnormal climate events exhibit an upward trend from 1990 to 2020.

The remainder of this paper is organized as follows. In the next section, the experimental results are described. Then, the conclusions, limitations, and future works are provided. Finally, the methodology of entropy-based dynamic graph embedding for detecting abnormal climate event is detailed.

## Results

Since the graphs with similar neighbor structures may have significantly different entropies, only using the adjacency matrices of the graphs cannot detect the anomaly. Therefore, we discriminate the anomaly by using the EDynGE model. To detect the anomaly in the embedding space, we applied the existing outlier detection methods, which are local outlier factor (LOF)^8^, isolation forest (IF)^9^, and box-plot (BP)^10^. IF method mentions that the distribution of outliers is sparse, and these outliers are far away from the normal observations with high density. Thus, the outliers can be easily separated. LOF detects outliers based on the density of the data points. The BP method is based on statistical indices for detecting outliers and requires the dataset to have a normal distribution.

### Datasets

For evaluating the proposed model, we utilized two kinds of datasets to conduct the experiments. Firstly, the performance of the proposed model is obtained by using synthetic climate data. Then, we applied the proposed model on the real-world daily climate data. The synthetic datasets are generated by using the LARS-WG weather generator that is a stochastic weather generator^11^. It can simulate the climate scenarios based on global or regional climate models and comprises the climate projections from the coupled model intercomparison project 5 (CMIP5)^12^. In this study, we utilized the datasets generated from 5 different institutions in the CMIP5 to conduct the experiments, which are Australian community climate and earth-system simulator model (ACCESS), Beijing climate center and China meteorological administration (BCC), Canadian center for climate modeling and analysis (CanCm4), Euro Mediterranean climate change Center (CMCC), and national meteorological research center (CNMR). Each dataset includes four-time series in 100 years, namely precipitation, maximum temperature, minimum temperature, and radiation.

The real-time daily climate data from the Chinese surface stations of ten provinces were used to conduct experiments. According to the nationwide surface climate statistical method^13^, these datasets were derived from various provincial meteorological bureaus through statistical compilations. The datasets were collected from 194 basic and reference surface meteorological observation stations and automatic weather stations in China from 1951. Each dataset included 18 elements, including mean pressure, mean temperature, precipitation and so on. In this study, we collected meteorological data from 1990 to 2020 to evaluate the EDynGE model.

### Evaluation metrics

Because the datasets are unlabeled, we propose using two different ways to evaluate the EDynGE model. The first way is to label a certain number of data points as outliers. These data points are embedded vectors of climate events. Since there are 1–10% anomalies in a dataset^14^, we conduct the experiments to validate the performance of the proposed model by selecting 1–10% anomalies. For example, we assume that 10% of data points are selected as outliers in each dataset. The embedding vector of the $t-{th}$ graph is denoted as $g_t$. The center of the embedding vectors can be formulated as $c = \frac{1}{T} \sum _{t=1}^{T}g_t$, where *T* is the number of time intervals. If the entropies of the graphs are similar, the embedding vectors of these graphs are close to each other, and the outliers are far from the normal observations. The 10% data points farthest from the center are selected as outliers. The EDynGE model can be evaluated using precision, recall, and F1-score.

In the second way, we propose a hypothesis based on global warming that with increasing temperature, the number of days of abnormal climate events also increases. We counted the number of days with abnormal climate events every year, every 5 years, and every decade. If the number of days of abnormal climate events detected by using our model exhibit an upward trend, it indicates that the proposed model is possible to detect the abnormal climate events.

### Baselines

To conduct a comparison, we utilized a graph convolutional neural network (GCN) and a dynamic graph to a vector-based model (dyngraph2vec) as baselines^15,16^. The GCN uses convolutional kernels to capture the spatial information of vertices in the graph. Because GCN is applied to the static graph, it does not consider the temporal information of the dynamic graph. Dyngraph2vec is an unsupervised learning model for embedding dynamic graphs. It provides the twp dyngraph2vec-based models, which are autoencoders (Dyn2vecAE) and the autoencoder-based recurrent neural network model (Dyn2vecAERNN). Dyn2vecAE cannot extract the temporal information from the dynamic graph since the model computes the embedding vectors by reconstructing the graphs. Dyn2vecAERNN utilizes the idea of the skip-gram to consider the temporal information of the graphs^17^. It computes the embedding vector of the current graph by using the graphs around the current graph.

### Results on synthetic data

**Figure 2:**
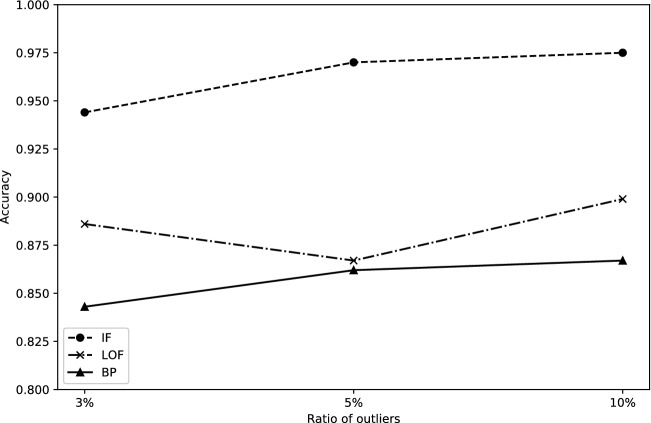
Performance for the dataset of BCC.

To obtain the performance of the proposed method with different ratios of anomalies, we select the ratio of anomalies from 1 to 10% to conduct the experiments, and the results are shown in Fig. 2. According to the results, the LOF method achieved the best F1-score and precision. The BP method showed an upward on the precision for the dataset of BBC. Since the BP method detects anomalies based on the distribution of the datasets, if the number of anomalies selected in the datasets is low, the BP method’s precision is low, and the recall of the BP method is high. With the increase of the number of anomalies, the recall of the BP method showed a downward trend, and the precision has an upward trend. LOF and IF methods can control the ratio of anomalies detected so that the performance of these two methods are not affected by selecting the different ratio of anomalies. Overall the performance for the dataset of BCC, LOF method performed better than the other two methods on precision and BP method exhibit a non-linear increase tend.

Since the ratio of anomalies is not more than 10% data points, we selected 10% of data points as anomalies in the synthetic dataset to evaluate the proposed model. Table 1 shows the comparison results on the synthetic climate data with respective to F1-score. According to the results, the EDynGE-based LOF method achieved the best F1-score on all synthetic climate datasets. The reason is that the proposed dynamic graph embedding model considered the similarity between two graphs on the entropy so that the graph with an abnormal graph was far from most normal observations. In this case, the graphs nearby the anomalies are less than the normal graphs, and the density of the anomalies is low. Therefore, the LOF method achieved a better F1-score than the other two methods. Overall, the BP method achieved the worst F1-score. Because the BP method requires that the dataset has a normal distribution and the dataset is not a normal distribution, the BP method cannot perform better than the other two methods.

**Table 1:** Comparison results on synthetic datasets (the bold scores indicate the best performance with the given datasets).

Datasets	EDynGE			Dyn2vecAE			GCN			Dyn2vecAERNN		
	LOF	IF	BP	LOF	IF	BP	LOF	IF	BP	LOF	IF	BP
ACCESS	**1**	0.74	0.55	0.89	0.70	0.48	0.98	0.88	0.34	0.93	0.92	0.53
BCC	**0.98**	0.76	0.67	0.94	0.80	0.73	0.97	0.78	0.60	0.78	0.80	0.62
CanCM4	**1**	0.78	0.62	0.98	0.92	0.68	0.86	0.89	0.61	0.86	0.90	0.56
CMCC	**0.99**	0.80	0.58	0.94	0.81	0.60	0.81	0.99	0.81	0.87	0.85	0.78
CNMR	**1**	0.75	0.67	0.94	0.52	0.43	0.95	0.82	0.56	0.95	0.41	0.61

### Results on real-world data

**Figure 3:**
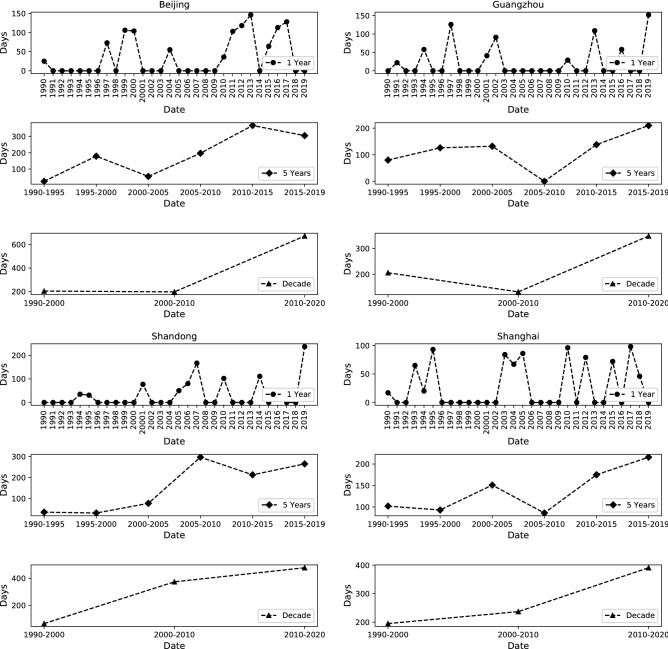
Days of abnormal climate events in the four provinces.

Figure 3 shows the days of abnormal climate events in the four provinces obtained using the IF method. The results of every year show that the frequency of abnormal climate events exhibits an increasing trend. We calculated the days of abnormal climate events every 5 years. The results showed that four provinces exhibited a non-linear increasing trend. However, a local minimum value in Guangzhou and Shanghai was observed from 2005 to 2010, and Beijing had a local minimum value from 2000 to 2005. The results of every decade indicated that three provinces Beijing, Shandong, and Shanghai showed an upward trend. Guangzhou showed a falling trend first followed by a rising trend. According to the experimental results, the detected abnormal climate events conform with the hypothesis in most cases.

Table 2 shows the F1-score of the models under 10% outliers. According to the experimental results, the EDynGE model exhibited the best performance in seven provinces, of which the EDynGE-based LOF method has the best performance in six provinces. The F1-score of the proposed method performed worse than the DynvecAE method on Jiangsu, Nei Mongol, and Shanxi provinces. The DynvecAE method utilized two auto-encoders to map the dynamic graph into an embedding space. Therefore, two graphs with a similar neighbor structure are close to each other in the embedding space. In this case, the abnormal graph is scattered and can be correctly detected. The proposed method clustered the graph based on their entropy. The graphs with the similar graph are far from the most observations but not scattered. Therefore, if the entropy of the abnormal graphs is similar, the performance of the proposed method is affected. The anomalies in Jiangsu, Nei Mongol, and Shanxi provinces have similar patterns, so the proposed method cannot outperform the DynvecAE method. In addition, based on the experimental results of the synthetic datasets and real-world datasets, the baselines have significant differences in the BP method. According to the results, the F1-score of the synthetic datasets performed better than the real-world datasets. The reason is that the synthetic datasets consist of four-time series that are much less than the real-world datasets. Therefore, with the increase of the number of time series, the performance of the BP method shows a downtrend. But the proposed method can overcome this problem by obtaining the experimental results.

**Table 2:** Comparison results on real-world meteorological datasets (the bold scores indicate the best performance with the given datasets).

Dataset	EDynGE			DynvecAE			GCN			Dyn2vecAERNN		
	LOF	IF	BP	LOF	IF	BP	LOF	IF	BP	LOF	IF	BP
Beijing	**1**	0.92	0.56	0.97	0.76	0.22	0.59	0.43	0.36	0.97	0.68	0.22
Shanghai	**0.97**	0.77	0.59	0.76	0.85	0.25	0.50	0.74	0.34	0.90	0.64	0.26
Guangzhou	**0.97**	0.85	0.51	0.93	0.78	0.13	0.83	0.72	0.85	0.79	0.65	0.21
Shandong	**0.97**	**0.97**	0.70	0.76	0.84	0.52	0.69	0.34	0.55	0.93	0.60	0.13
Hebei	**1**	0.67	0.70	0.97	0.72	0.46	0.57	0.50	0.11	0.97	0.72	0.11
Jiangsu	0.92	0.71	0.60	**0.93**	0.85	0.26	0.43	0.13	0.14	0.71	0.70	0.25
Nei Mongol	0.88	0.70	0.25	0.60	**0.92**	0.26	0.33	0.16	0.14	0.58	0.69	0.41
Shanxi	0.69	0.65	0.56	0.61	**0.73**	0.59	0.39	0.25	0.33	0.96	0.59	0.41
Zhejiang	0.67	**0.81**	0.67	0.33	0.62	0.29	0.59	0.74	0.26	0.50	0.67	0.26
Tianjin	0.62	**0.77**	0.52	0.71	0.80	0.60	0.67	0.50	0.21	0.88	0.63	0.25

**Figure 4:**
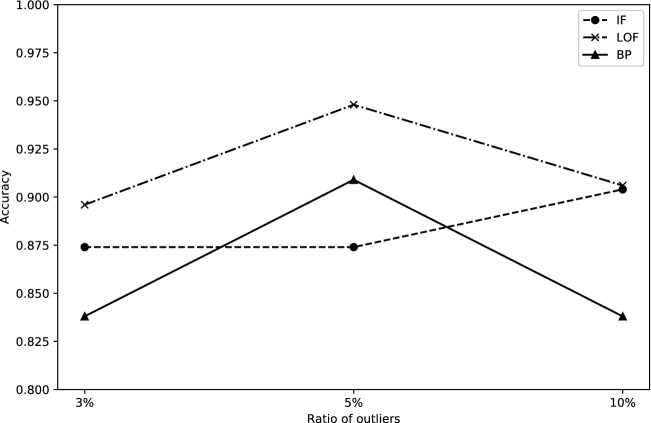
Performance for the city of Beijing.

In the real-world climate data, we obtained the performance of the EDynGE model with different ratios of outliers. Figure 4 shows the performance of the model on Beijing province by choosing different ratios of outliers. According to the results, the LOF method achieved the best performance on the ratio of 1%, 2%, and 10% with the F1-score of 1. When we selected 1% and 2% anomalies, these data points are most far from the center of the embedding space, and the density of these points is low so that the LOF method can exhibit the best performance on these two ratios. With the ratio of anomalies increasing, some of the normal data points far from the center are wrongly detected as the outlier. Therefore, the performance of the LOF method is affected. As the ratio of anomalies continues to increase, these points are labeled as anomalies. Therefore, the performance of the LOF method showed an upward trend after a downward trend. The F1-score of the IF method is more stable than the LOF method, but the IF method’s maximum value is lower than the LOF method. The performance of the BP method also showed an upward trend that is the same as the synthetic data. In addition, the precision of the LOF method is better than the other two methods. Overall, the conclusion of experimental results for the dataset of synthetic data and real-world data are the same, which LOF method outperformed the other two methods with respect to F1-score.

## Discussion

In this study, we proposed an EDynGE model to detect abnormal climate events. The model uses the spurious correlation coefficient to calculate the graph entropy and reduces the distance between two climate events with similar graph entropy. We conducted experiments to validate the performance and stability of the EDynGE model for abnormal climate event detection. The results showed that the LOF method exhibited better results than the IF and BP methods by 12.3% and 66.2% with respective to F1-score, respectively. The EDynGE model performed better than the other dynamic graph embedding models by 43.2% with respective to F1-score. Based on global warming, we hypothesized that with an increase in temperature, the number of days of abnormal climate events exhibits an upward trend. The experimental results showed that the number of abnormal climate event days increased by 304.5 days from 1990 to 2020, which agreed with the hypothesis. This indicates that the EDynGE model can detect abnormal climate events. According to the experimental result, the proposed model achieved the same conclusions on the synthetic datasets and real-world meteorological datasets.

This study has some limitations. The EDynGE model can cluster graphs based on graph entropy. However, graphs with different neighbor structures have the same graph entropy in some cases. The EDynGE model cannot detect outliers with an abnormal neighbor structure. This indicates that the EDynGE model ignores the spatial information of the dynamic graph. To overcome this issue, we plan to construct a hybrid model that consists of the neighbor structure similarity and graph entropy similarity for detecting outliers in multiple time series. The second limitation is that the EDynGE model is based on an autoencoder that does not capture the temporal information from the dynamic graph. Although the temporal information is negligible in the research problem, it also needs to be considered in dynamic graph embedding. To overcome this problem, we plan to construct an autoencoder model using the long short-term memory architecture to discover temporal features from the dynamic graph.

We propose a novel idea to detect outliers from multiple time series. It utilizes the correlation of the time series to construct a dynamic graph and detects the outlier from the dynamic graph. The outlier detection problem is transformed from the multiple time series domain to the dynamic graph domain. It can help people find the causes of the outliers by obtaining the evolution of graphs. For example, abnormal trends in the stock market can be detected and analyzed using the EDynGE model in the financial time series. Furthermore, the digital twin technology is developing rapidly. It utilizes sensors to record the digital information for simulating the condition of the object in the physical space. The proposed is able to detect the anomalies from the recorded digital signals to diagnose faults from the physical. For example, the transmission failure in the machines and the structural damage in the buildings can be detected by using the proposed idea.

## Methods

This section describes the dynamic climate graph, graph entropy, and EDynGE model. A dynamic graph is used to model climate events in each time interval to detect the abnormal climate event. The graph entropy measures information regarding the climate event. The meteorological datasets are allowed to collect from the China meteorological data service center (http://data.cma.cn/en) by registering an account. The proposed consists of three processes. Firstly, the dynamic graph is constructed by identifying the spurious relationship between the time series and compute the graph entropy. Then, to discriminate the anomalies, we propose a novel dynamic graph embedding model based on the graph entropy. Finally, the anomalies are detected by applying the existing outlier detection methods.

### Graph construction

The graph is denoted as *G*(*V*, *E*), where *V* and *E* denote the vertices and edges, respectively. For a climate event, the vertex indicates the meteorological data, the edge denotes the spurious relationship, and the weight *w* indicates the spurious correlation coefficient. The coefficient is calculated based on the causality and correlation between two time series, *x* and *y*. The time series causality is defined as follows.

#### Definition 2

(*Time series causality*) The causality of two series is defined as that if one of the series improves the prediction of the other, which is formulated as

1 $$\begin{aligned} C(x,y) = \left\{ \begin{array}{rll} 1 &{}\quad if \, p \ge 0.05&{} \\ 0 &{}\quad otherwise&{} \\ \end{array}\right. \end{aligned}$$

where *x* and *y* are two time series, *C*(*x*, *y*) indicates the causality between them, and *p* is the probability that the two series are not causally related.

The Granger causality test is utilized to calculate the short-run causality between two meteorological time series, *x* and *y*^18^. The test makes a null hypothesis that the two series are not causally related and includes two predictions. Firstly, it uses the past values of the series *y* as variables to predict the current *y*. Then, it uses the past values of series *x* and *y* as variables to predict the current *y*. If the prediction result obtained using the temporal information of two series *x* and *y* is better than the prediction only using the series *y*, then *x* helps predict *y*. The t-test was utilized to compare the difference between two prediction results^19^. The *p* value was used to denote the probability of the null hypothesis. If the *p* value was more than 0.05, then the two series *x* and *y* were said to not be causally related^20^.

The weight value of an edge in the graph indicates the spurious correlation coefficient that is calculated using the causality and Pearson correlation coefficient (PCC)^21^, which is formulated as follows.

2 $$\begin{aligned} R(x,y) = \left\{ \begin{array}{lll} 1 &{}\quad if \, C(x,y) = 0&{} \\ C(x,y)-|PCC| &{}\quad otherwise&{} \\ \end{array}\right. \end{aligned}$$

where *C*(*x*, *y*) indicates the causality between the two series *x* and *y*. *R*(*x*, *y*) indicates the spurious correlation coefficient between the two series. The spurious correlation coefficient is inversely proportional to the causality between the time series *x* and *y*. If the causality *C*(*x*, *y*) between two series *x* and *y* is 0, then there is no spurious correlation between the two series. In this case, the corresponding spurious correlation coefficient *R*(*x*, *y*) is 1.

### Graph entropy

The graph entropy is calculated based on information entropy^22^. We assume that there are two independent events, *x* and *y*. The information of these events should be satisfied as $h(x,y) = h(x) + h(y)$, where *h*(*x*) indicates the information of the event *x*, and *h*(*x*, *y*) indicates the information of these two events occurring at the same time. The probability of these events should be satisfied as $p(x,y) = p(x)\times p(y)$, where *p* indicates the probability of the event. The information of the event *x* can be measured as $h(x) = -log_2p(x)$. Information entropy can be represented as the information of the event *x* times the probability of *x*, which is formulated as $e(x) = -p(x)log_2p(x)$. For a set of events *X*, the information entropy is formulated as $e(X)=-\sum _{i=1}^{N}p(x_i)log_2p(x_i)$, where *N* indicates the number of events in the set and $x_i$ indicates the *i*-*th* event.

To calculate the graph entropy, we calculated the entropy for each vertex in the graph. The definition of the vertex entropy is defined as follows.

#### Definition 3

(*Vertex entropy*) Given a graph $G=(V,E)$, the entropy of the vertex $v_i$ is defined based on the weight between the vertices $v_i$ and $v_j$, which is formulated as $e(v_i) = \sum _{j=0,j \ne i}^{N}-w_{i,j}log_2w_{i,j}$, where *N* indicates the number of vertices. The weight value $w_{i,j}$ equals $R(v_i,v_j)$, which denotes the spurious correlation coefficient between two vertices $v_i$ and $v_j$.

The graph entropy is calculated by summing the entropy of all vertices, which is formulated as $e(G) = \sum _{i=0}^{N}e(v_i)$. The dynamic graph entropy is composed of the graph at time interval $t \in [0,T]$, which is formulated as ${\mathscr {E}} = \{e(G_t)|t\in [0,T]\}$. The information of the climate event can be quantified using graph entropy. When one of the meteorological data points changes, the spurious relationship coefficients change, and the graph entropy also changes at the corresponding time interval. The abnormal climate event can be detected by obtaining graph entropy.

### Entropy-based graph embedding

The dynamic graph consists of graphs $G_t$ in the time interval $t \in [0,T]$, which is formulated as ${\mathscr {G}} = \{G_t|t\in [0,T]\}$. Dynamic graph embedding is used to capture the temporal information of the dynamic graph ${\mathscr {G}}$ for learning a mapping function $f: G_t \rightarrow g_t$, where $g_t$ is an embedding vector of the graph $G_t$. The similarity of the entropy between the two graphs is formulated as $d(e(G_i),e(G_j)) = \sqrt{||e(G_i) - e(G_j)||^2_2}$. The object of the entropy-based graph embedding reduces the distance between two graphs with similar entropy. Therefore, for one graph, we have to find a corresponding graph most similar to it. Then, we reduce the distance between these two graphs in the embedding space by establishing the loss function. For example, given a graph $G_i$ at the *i*-*th* time interval, we can find a graph $G_j$ at the *j*-*th* time interval that is the most similar graph with $G_i$. Since the graphs $G_i$ and $G_j$ has a similar entropy, the embedding vectors $g_i$ and $g_j$ have a short distance in the embedding space by minimizing the loss function. To address this problem, we construct a dynamic supervised graph, which is defined as follows.

#### Definition 4

(*Dynamic supervised graph*) Let ${\mathscr {G}} = \{G_t|t\in [0,T]\}$ denote the dynamic graph at time interval $t\in [0,T]$. For the graph $G_t$, the corresponding graph $G_i$ can be found from the dynamic graph ${\mathscr {G}}$, where the similarity $d(e(G_i),e(G_t))$ of the entropy between the two graphs is the smallest. The supervised graph is defined as the corresponding graph $G_i$ which is denoted as $S_t = \mathop {\arg \min }\limits _{G_i}(d(e(G_i),e(G_t)))$. The dynamic supervised matrix is a set composed of the graph $G_i$, which is formulated as ${\mathscr {S}} = \{S_t|t\in [0,T]\}$.

**Figure 5:**
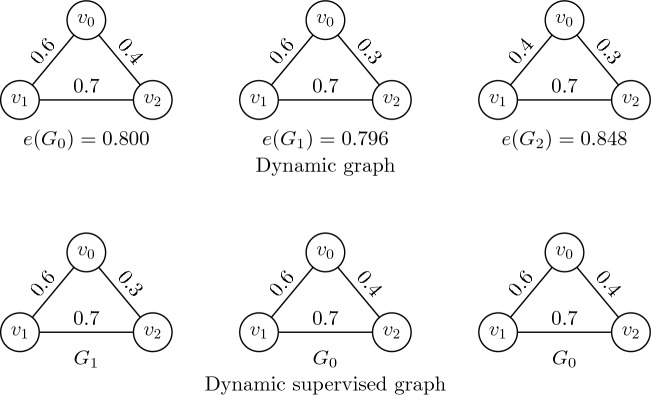
Example of the dynamic supervised graph.

As shown in Fig. 5, the dynamic graph is formulated as ${\mathscr {G}} = \langle G_0, G_1, G_2 \rangle$. The entropy of vertex $v_0$ in the climate event $G_0$ is calculated as $e(v_0) = \sum _{j=0,j \ne i}^{2}-w_{0,j}log_2w_{0,j} = -0.6\times log_2 0.6 - 0.4\times log_2 0.4 = 0.292$. The entropies of the vertices $v_1$ and $v_2$ in $G_0$ can be calculated as $e(v_1) = 0.241$ and $e(v_2) = 0.267$, respectively. The entropy of graph $G_0$ is $e(G_0) = 0.292 + 0.241 + 0.267 = 0.800$. The entropy of graphs $G_1$ and $G_2$ are $e(G_2) = 0.796$ and $e(G_2) = 0.848$, respectively. The similarity of the entropy between the graphs $G_0$ and $G_1$ is $d(e(G_0),e(G_1)) = 0.004$. The similarity between the graphs $G_0$ and $G_2$ is $d(e(G_0),e(G_2)) = 0.048$. Therefore, the nearest graph from $G_0$ is $G_1$, the nearest graph from $G_1$ is $G_0$, and the nearest graph from $G_2$ is $G_0$. The dynamic supervised matrix is thus denoted by ${\mathscr {S}} = \langle G_1, G_0, G_0 \rangle$.

**Figure 6:**
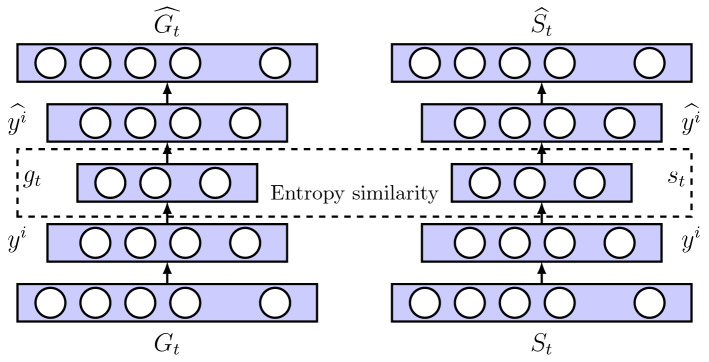
Architecture of EDynGE model.

We utilize two autoencoders to reconstruct the dynamic graph and supervised graph. The two autoencoders share parameters with each other. Figure 6 shows the architecture of the EDynGE model, where $G_t$ and $S_t$ indicate the climate graph and the supervised graph, respectively. The embedding vectors of $G_t$ and $S_t$ are denoted as $g_t$ and $s_t$, respectively. The autoencoder includes an encoder and decoder. We use $y^i$ to indicate the *i*-*th* layer of the encoder, and $\widehat{y^i}$ is used to denote the *i*-*th* layer of the decoder. The autoencoder reconstructs the input data using the encoder and decoder to calculate the graph’s embedding vector. The encoder uses non-linear functions to extract the features for mapping the graphs into the embedding space, which are formulated as

3 $$\begin{aligned} y^1= & {} \delta (W^1G_t+b^1)\end{aligned}$$

4 $$\begin{aligned} y^i= & {} \delta (W^iy^{i-1}+b^i) \end{aligned}$$

where $\delta$ is an activation function. $W^i$ and $b^i$ indicate the weight and basis in the *i*-*th* layer, respectively. The ReLU function is utilized as the activation for making the neural network non-linear, which is formulated as $f(y^i)=max(0,y^i)$^23^. The decoder reconstructs the graph from the embedding vector, which is calculated by reversing the encoder’s computation.

The purpose of dynamic embedding is to reduce the distance between two graphs that have a similar entropy in an embedding space. Therefore, we establish a loss function based on the similarity of the graph entropy in the embedding layer, which is formulated as ${\mathscr {L}}_s = \frac{1}{T}\sum _{t=1}^{T}||g_t - s_t||^2_2$, where *T* indicates the number of time intervals. The graph $G_t$ and supervised graph $S_t$ have the smallest similarity on graph entropy. Thus, the function ${\mathscr {L}}_s$ reduces the loss between $a_t$ and $s_t$ to reduce the distance between two graphs in the embedding space. An autoencoder is used to reconstruct the input so that we have to establish a loss function for reducing the loss between the input and output, which are formulated as ${\mathscr {L}}_1 = \frac{1}{T}\sum _{t=1}^{T}||G_t - \widehat{G_t}||^2_2$. To avoid overfitting, we establish a regularization term that is formulated as ${\mathscr {L}}_{reg} = \frac{1}{2}\sum _{i=0}^{I}(||W^i||_2^2 + ||\widehat{W^i}||^2_2)$, where $W^i$ and $\widehat{W^i}$ indicates the weight of the encoder and decoder in the *i*-*th* layer. The joint loss function is established using the functions ${\mathscr {L}}_s$, ${\mathscr {L}}_1$, and ${\mathscr {L}}_{reg}$, which is formulated as

5 $$\begin{aligned} {\mathscr {L}} = \frac{1}{T}\sum _{t=1}^{T}||g_t - s_t||^2_2 + \frac{1}{T}\sum _{t=1}^{T}||G_t - \widehat{G_t}||^2_2 + \frac{1}{2}\sum _{i=0}^{I}(||W^i||_2^2 + ||\widehat{W^i}||^2_2) \end{aligned}$$

We utilize the gradient descent algorithm and backward propagation algorithm^24,25^ to train the model. Gradient descent is used to calculate the weight and basis in the output layer, which are formulated as $W^I = W^I - \eta \frac{\partial {\mathscr {L}}}{\partial W^I}$ and $b^I = b^I - \eta \frac{\partial {\mathscr {L}}}{\partial b^I}$, where *I* indicates the output layer. Each layer’s weight and basis are calculated using the backward propagation algorithm, which calculates the partial derivation of the loss function based on the chain rule for updating each layer’s weight and basis.

## Data Availability

The real-meteorological datasets used in this study are available to be collected from the China Meteorological Data Service Center and allow the authors to download the data from the website http://data.cma.cn/en by registering an account.
